# Superior Mesenteric Artery Syndrome in a 6-Year-Old Girl with Final Diagnosis of Celiac Disease

**DOI:** 10.1155/2019/3458601

**Published:** 2019-04-08

**Authors:** F. Salehzadeh, Ali Samadi, M. Mirzarahimi

**Affiliations:** ^1^Pediatric Rheumatology, Bou-Ali Children's Hospital, Ardabil University of Medical Sciences (ARUMS), Ardabil, Iran; ^2^Bou-Ali Children's Hospital, Ardabil University of Medical Sciences (ARUMS), Ardabil, Iran

## Abstract

**Background:**

Superior mesenteric artery syndrome (SMAS) occurs when the duodenum is compressed between the two arteries, superior mesenteric artery and aorta. The complications of this rarely found disorder in children range from causing trouble in duodenal functions to intestinal obstruction which is potentially life-threatening.

**Case Presentation:**

Here we are reporting a case of SMAS in a 6-year-old girl with the complaint of chronic abdominal pain since 3 years. She suffered from growth failure, while different workups were negative. Ultimately, imaging investigations detected superior mesenteric artery syndrome as an etiologic background. In the additional investigations, it is found that she suffered from celiac disease.

**Conclusion:**

We concluded that the inflammatory nature of the celiac disease can affect the anatomy of the duodenum beyond its mucosal surface in the mesenteric fat tissue and results in SMAS.

## 1. Introduction

Superior mesenteric artery (SMA) syndrome due to its obstructive nature of the intestine is counted a serious disease. Although superior mesenteric artery syndrome is rare, its burden-associated complications make it a crucial differential diagnosis for bowel obstruction, especially in the setting of recent weight loss [1]. It results from compression of the duodenum between the abdominal aorta posteriorly and the superior mesenteric artery anteriorly, which is thought to be related to the loss of the duodenal fat pad [2]. The symptoms typically are nonspecific gastrointestinal complaints including nausea, vomiting, chronic intermittent abdominal pains, early satiety, and anorexia [3]. Mainly it affects adolescents and young adults and rarely children. We are reporting a preschool aged girl with failure to thrive (FTT) and chronic abdominal pain associated SMA syndrome and final diagnoses of celiac disease.

## 2. Case Presentation

A 6-year-old girl with the complaint of vague periodic abdominal pains mainly in the epigastrium and periumbilical region is referred to the children's hospital. There was not any correlation with feeding and almost having discomfort at night times. She had periods of constipation and diarrhea and symptoms of leg pains. She also used to take Propranolol for early morning headaches mostly in the frontal region for 2 years. The patient with a probable diagnosis of Familial Mediterranean Fever (FMF) was referred to the pediatric rheumatology clinic for further surveys when 0.5 milligram daily colchicine was taken without considerable clinical response. She also had both an intussusception experiment and a history of negative appendectomy since she was 2 years old and the initiation of abdominal pain since that time. She was never febrile during abdominal pains. General examination revealed a thin hemodynamically stable girl with no signs of acute abdomen; the weight and the length were 15 kg and 105 cm [body mass index (BMI): 14.5 kg/m^2^].

**Figure 1:**
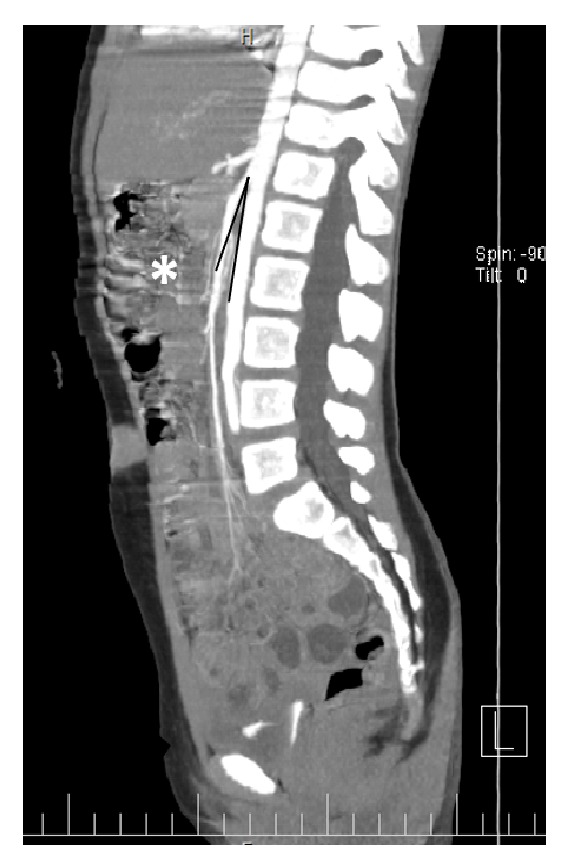
Contrast-enhanced sagittal reconstruction CT scan of superior mesenteric artery (SMA) and its adjacent anatomy shows narrowing of the aortomesenteric angle to less than 15° and distended stomach (*∗*) and full of fluids loops of the small intestine.

She had second admission within previous 15 days with similar complaints. The grey scale and color Doppler abdominal ultrasonography showed normal portal and splenic veins and there were no evidences of thrombosis. Stool calprotectin test was also requested for a probable inflammatory condition in the gastrointestinal tract. However, because of persistent symptoms, abdominal CT angiography was obtained, which revealed a decrease in aortic and superior mesenteric artery and aortomesenteric angle consequently to 5 mm and 14 degrees suggestive for superior mesenteric syndrome along with a gastric and duodenal dilation [Figure 1].

Another confirmatory spiral CT scan of abdominopelvic region also marked distended stomach and the proximal part of duodenum which in context of CT angiography proposing SMA syndrome. In addition, she was in a malnourished state without adequate evidences supporting an autoimmune condition. Thus, according to persistent symptoms and FTT, we checked serology of the celiac disease, antigliadin, and anti t-transglutaminase antibodies, which showed positive serologic results and were confirmed by pathologic study. Following the final diagnoses of Gluten-sensitive enteropathy, she gradually gained weight and growth indexes improved, and the obstructive symptoms resolved by taking Gluten-free diet in a 6-month regular follow-up period by the pediatrician.

## 3. Discussion 

The superior mesenteric artery syndrome (SMAS) is a rare form of upper gastrointestinal obstruction diseases, defined as a vascular compression of the third part of the duodenum between the superior mesenteric artery (SMA) and the aorta [4]. It results in chronic, intermittent, or acute complete or partial duodenal obstruction [5]. The true incidence of this syndrome is unknown, but it has been estimated to be approximately 0.013–0.3% among general population [6], although it is much rarer in children.

SMAS diagnosis should be noted particularly if the symptoms persist and the common causes of childhood abdominal pains and vomiting such as acute viral gastroenteritis, food poisoning, and gastritis have been ruled out.

Delay in the diagnosis of superior mesenteric artery syndrome can result in malnutrition, dehydration, electrolyte abnormalities, gastric pneumatosis, portal venous gas formation, hypovolemia secondary to massive GI hemorrhage, and even death secondary to gastric perforation [7].

This uncommon condition in children results from high catabolic states and malnutrition by reducing retroperitoneal fat pads and secondary angle changes between superior mesenteric artery and the duodenum consequently [8]. Patients typically present with abdominal pain, distension, tenderness, and vomiting. Other symptoms include abdominal hypersensitivity, weight loss, diarrhea, gastric reflux, feeding difficulties, and early satiety [9].

Severe burns, malignancy, anorexia nervosa, malabsorption, and hypermetabolic states are the predisposing conditions mentioned in SMAS [1, 9].

The diagnosis of SMA syndrome is primarily based on clinical suspicion. However, imaging is necessary for confirmation [10] by the loss of an angle between the SMA and the abdominal aorta to less than 20 degrees. The distance between the two vessels is also decreased to less than 6 mm (the normal distance is 8–12 mm) [11].

Conservative management and surgical bypass of the obstruction are therapeutic options for SMA syndrome. The goal of ultimate medical therapy is to induce weight gain, which would presumably result in an increase in fat pad at the mesenteric root [9].

The probable association of celiac disease and SMAS has already been reported in a 7-year-old boy with a history of a Deloyers procedure for subtotal colonic Hirschsprung. Three years after surgical operation because of progressive weight loss and falling of the growth indexes, a comprehensive workup revealed celiac disease [4].

In case of failure in conservative treatment surgical intervention should be planned; a duodenojejunostomy remains the operation of choice to relieve the obstruction, with success rates of up to 90% [6]. However, we observed clinical improvement in our case by Gluten-free diet during follow-up, and because of her weight gain and prevention of overexpose to radiation we did not evaluate second CT-angioscan.

## 4. Conclusion

Although SMA syndrome is not a common condition in pediatrics, it should be considered as a rare cause of abdominal pain in children with celiac disease.

## Data Availability

The author can be contacted for data requests.
